# Triterpenes from *Poria cocos* suppress growth and invasiveness of pancreatic cancer cells through the downregulation of MMP-7

**DOI:** 10.3892/ijo.2013.1902

**Published:** 2013-04-16

**Authors:** SHUJIE CHENG, ISAAC ELIAZ, JUNFANG LIN, DANIEL SLIVA

**Affiliations:** 1 Department of Bioengineering, College of Food Science, South China Agricultural University, Guangzhou, P.R. China;; 2 Cancer Research Laboratory, Methodist Research Institute, Indiana University Health;; 3 Department of Medicine, School of Medicine, Indiana University, Indianapolis, IN;; 4 Amitabha Medical Clinic and Healing Center, Sebastopol, CA, USA

**Keywords:** triterpenes, *Poria cocos*, pancreatic cancer, pachymic acid, MMP-7

## Abstract

*Poria cocos* is a medicinal mushroom that is widely used in traditional Asian medicine. Here, we show that a characterized mixture of triterpenes extracted from *P. cocos* (PTE) and three purified triterpenes: pachymic acid (PA), dehydropachymic acid (DPA) and polyporenic acid C (PPAC) suppress the proliferation of the human pancreatic cancer cell lines Panc-1, MiaPaca-2, AsPc-1 and BxPc-3. Moreover, the most effective compound, PA, only slightly affects the proliferation of HPDE-6 normal pancreatic duct epithelial cells. The anti-proliferative effects of PTE on BxPc-3 cells are mediated by the cell cycle arrest at G0/G1 phase. DNA microarray analysis demonstrated that PTE significantly downregulates the expression of *KRAS* and matrix metalloproteinase-7 (*MMP-7*) in BxPc-3 cells. In addition, PTE and PA suppress the invasive behavior of BxPc-3 cells. The inhibition of invasiveness by PTE and PA was associated with the reduction of MMP-7 at the protein level and the role of MMP-7 further confirmed by the gene silencing of *MMP-7* which also suppressed the invasiveness of BxPc-3 cells. In conclusion, triterpenes from *P. cocos* demonstrate anticancer and anti-invasive effects on human pancreatic cancer cells and can be considered as new therapeutic agents in the treatment of pancreatic cancer.

## Introduction

Pancreatic cancer is the fourth leading cause of cancer-related deaths in the United States. As one of the most aggressive human cancers, the 5-year survival rate of pancreatic ductal adenocarcinoma (PDAC) is <5% (1). Recent studies suggested that bioactive compounds from mushrooms could protect against multiple myeloma, breast and skin cancer (2–5). *Poria cocos* (also known as *Wolfiporia extensa*) is a medicinal mushroom in the *Polyporaceae* family that grows in pine trees and its sclerotium is widely used in traditional Asian medicine for its sedative, diuretic, digestive and tonic effects (6–8). Although the anticancer activity of polysaccharides extracted from *P. cocos* is associated with the stimulation of immune response and these polysaccharides significantly enhance immunopotentiation (9), triterpenes isolated from *P. cocos* have a direct inhibitory effect on cancer cells through a variety of mechanisms including inhibition of cell proliferation, induction of apoptosis and suppression of invasive behavior (10–16). However the effect of triterpenes from *P. cocos* against pancreatic cancer remains to be evaluated and the mechanism determined.

In the present study, we evaluated the effects of a triterpene mixture extracted from *P. cocos* (PTE) and three purified triterpenes: pachymic acid (PA), dehydropachymic acid (DPA) and polyporenic acid C (PPAC), on growth and invasive behavior of human pancreatic cancer cell lines Panc-1, MiaPaca-2, BxPc-3 and AsPc-1 and normal pancreatic duct epithelial cell line HPDE-6. PTE as well as PA, DPA and PPAC inhibit growth of pancreatic cancer cells and PTE and PA significantly suppress invasive behavior of BxPc-3 cells by inhibiting expression of MMP-7. Taken together, our results indicate that triterpenes from *P. cocos* may be potentially exploited for the use in pancreatic cancer intervention.

## Materials and methods

### Reagents

Dried sclerotium of *P. cocos* from Fujian, P.R. China was provided by Professor Zhonglin Yang (China Pharmaceutical University, Nanjing, P.R. China). It was authenticated by School of Traditional Chinese Medicine at China Pharmaceutical University. Voucher specimens were deposited at State Key Laboratory of Natural Medicines, China Pharmaceutical University. DMSO was purchased from Sigma (St. Louis, MO). All other chemicals and reagents were of analytical grade. Anti-MMP-7 and anti-β-actin antibodies were obtained from Santa Cruz Biotechnology (Santa Cruz, CA).

### Extraction and purification

**Figure 1:**
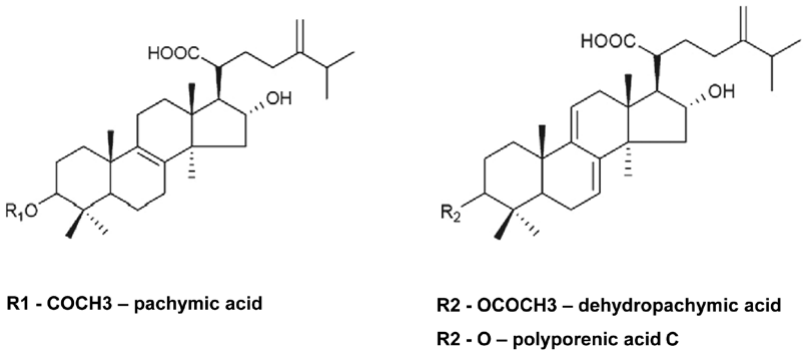
Structure of lanostane-type triterpenoids isolated from *P. cocos*.

Pulverized sclerotium of *P. cocos* (2.0 kg) was extracted three times with 95% ethanol (10 l) under reflux for 3 h at room temperature. The ethanol solution was combined and evaporated in vacuum to give a crude extract (38 g). The crude extract was mixed with silica gel G (size: 200–300 mesh) and fractionated on silica column chromatography by gradient elution using petroleum ether and ethyl acetate (100:0→75:15→1:1). Fractions were collected, combined and subjected to further chromatography on a silica gel→H (size: 60–120 mesh) column by step gradients of cyclohexane-ethyl acetate (100:0→75:15). The collected fractions were combined on the basis of their thin-layer chromatography (TLC) characteristics to give three pooled fractions: pooled extracts A, B and C (PEA, PEB and PEC), listed in increasing order of polarity. Part of the PEB (PTE) was subjected to high-performance preparative liquid chromatography (Daojing, Japan, model: SPD-20A), from which three pure compounds, PPAC, DPA and PA (Fig. 1) were isolated with isocratic elution of CH_3_OH-H_2_O (85:15), with trifluoroacetic acid added at 0.05%. Quantification of HPLC analysis demonstrated that PTE contains 55.7% PA, 31.7% DPA and 4.1% PPAC. Identification of PA, DPA and PPAC was conducted by comparison of their physical and spectroscopic data (^1^H-, ^13^C-NMR and MS) with the corresponding compounds reported in the literatures. PTE, PA, DPA and PPAC were dissolved in DMSO at a concentration of 50 mg/ml and 50 mM, respectively then stored at −20°C.

### Cell culture

The human pancreatic cancer cell lines Panc-1, MiaPaca-2, BxPc-3 and AsPc-1 were obtained from ATCC (Manassas, VA). Panc-1 cells were maintained in Dulbecco’s modified Eagle’s medium containing penicillin (50 U/ml), streptomycin (50 U/ml) and 10% fetal bovine serum (FBS). MiaPaca-2 cells were maintained in Dulbecco’s modified Eagle’s medium containing penicillin (50 U/ml), streptomycin (50 U/ml), 10% FBS and 2.5% horse serum (HS). BxPC-3 and AsPC-1 cells were maintained in RPMI-1640 medium containing penicillin (50 U/ml), streptomycin (50 U/ml) and 10% FBS. Media came from ATCC. Supplements, FBS and HS were obtained from Gibco BRL (Grand Island, NY). The normal human pancreatic duct epithelial cell line HPDE-6 was a generous gift from Dr Ming-Sound Tsao (University of Toronto, Toronto, Ontario, Canada). HPDE-6 cells were routinely cultured in keratinocyte serum-free (KSF) medium supplemented by epidermal growth factor and bovine pituitary extract. Medium and supplements came from Gibco BRL.

### Cell proliferation and cell viability

Human pancreatic cancer cells and normal human pancreatic duct epithelial cell line were treated with indicated concentrations of PTE, PA, DPA or PPAC for 24–72 h and cell proliferation determined as described (17). Cell viability was determined after incubation with PTE, PA, DPA or PPAC for 24 h by staining with trypan blue as described (18). Data are the mean ± SD from three independent experiments.

### Cell cycle analysis and invasive behavior assays

Cell cycle analysis of BxPc-3 cells incubated in the presence of PTE (0–5.0 *μ*g/ml) for 24 h was evaluated as described (19). Cell invasion of BxPc-3 cells treated with PTE (0–5.0 *μ*g/ml), PA (0–5.0 *μ*M) or transfected with *MMP-7* or control siRNA were performed as described (20). Data points represent the mean ± SD of three individual filters within one representative experiment repeated at least twice.

### DNA microarrays

BxPc-3 cells were treated with PTE (0, 5.0 *μ*g/ml) for 24 h and RNA isolated with RNeasy^®^ Mini Kit (Qiagen, Valencia, CA). RNA quality was monitored and quantified using the Qubit^®^ 2.0 Fluorometer (Invitrogen, Carlsbad, CA). Reverse transcription was performed with High Capacity cDNA Reverse Transcription Kit (Applied Biosystems, Foster City, CA) using 2.0 *μ*g total RNA. PCR analysis was performed on TaqMan^®^ Array Human Pancreatic Adenocarcinoma and 7900HT Fast Real-Time PCR System according to the manufacturer’s protocol (Applied Biosystems). Analysis of the relative quantity gene expression (RQ) data was normalized by *HPRT1* expression and was performed using the 2-^ΔΔCt^ method (21).

### Western blot analysis

BxPc-3 cells were treated with PTE (0–10 *μ*g/ml), PA (0–10 *μ*M), DPA (0–10 *μ*M) or PPAC (0–10 *μ*M) for 24 h. Whole cell extracts isolated from cells were prepared and western blot analysis with MMP-7 antibody was performed as previously described (17). Western blots were quantified with HP-Scanjet 550c and analyzed by UN-SCAN-IT software (Silk Scientific, Orem, UT).

### siRNA transfection

BxPc-3 cells were transfected with human *MMP-7* siRNA or control siRNA-A using siRNA Reagent System according to the manufacturer’s instructions at a final concentration of 60 nM (Santa Cruz, CA). After 48 h of transfection, the cells were harvested and *MMP-7* knockdown was verified by western blot analysis.

### Statistical analysis

Data are presented as mean ± standard deviation (SD). Statistical comparison between groups of data was carried out using ANOVA. P<0.05 was considered to be significant.

## Results

### Triterpenes from P. cocos suppress proliferation of human pancreatic cancer cell lines

**Figure 2:**
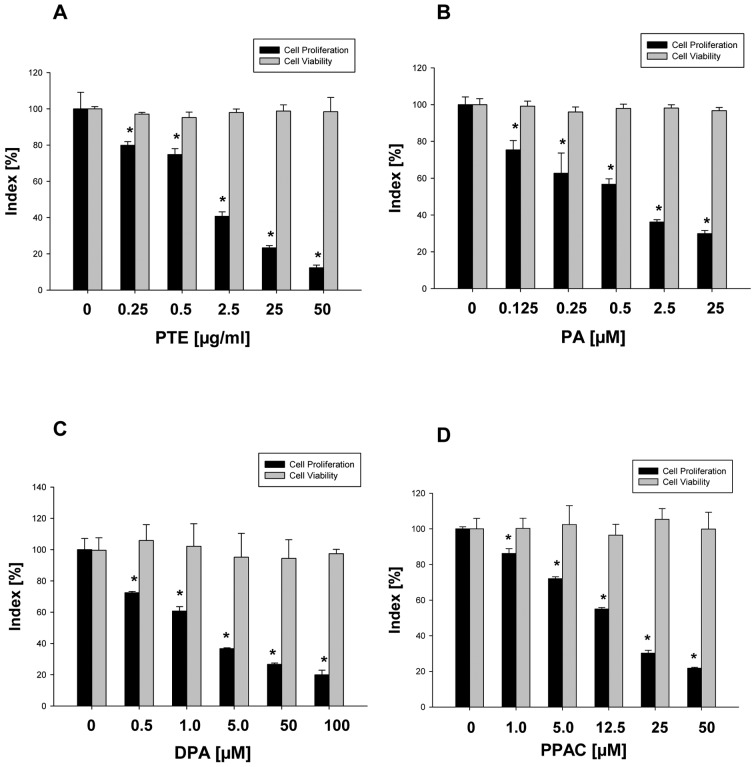
Effect of PTE, PA, DPA and PPAC on growth and viability of BxPc-3 cells. BxPc-3 cells were treated with (A) PTE (0–50 *μ*g/ml), (B) PA (0–25 *μ*M), (C) DPA (0–100 *μ*M) or (D) PPAC (0–50 *μ*M) for 24 h. Cell proliferation and viability were determined as described in Materials and methods. Each bar represents the mean ± SD of three experiments. Similar results were obtained in three independent experiments. Statistical analysis by ANOVA. ^*^P<0.05.

As previously demonstrated *P. cocos* triterpenes: PA, DPA and PPAC inhibited growth of human breast cancer, lung cancer and prostate cancer cells (11–13,22). To evaluate whether these triterpenes also affect growth of different human pancreatic cancer cell lines, Panc-1, MiaPaca-2, AsPc-1 and BxPc-3 cells were treated with PTE (0–80 *μ*g/ml) for 24 and 48 h and the proliferation determined as described in Materials and methods. PTE suppresses proliferation of Panc-1 (IC_50_-24 h = 28.3 *μ*g/ml, IC_50_-48 h = 24.5 *μ*g/ml), MiaPaca-2 (IC_50_-24 h = 29.4 *μ*g/ml, IC_50_-48 h = 23.0 *μ*g/ml), AsPc-1 (IC_50_-24 h = 13.7 *μ*g/ml, IC_50_-48 h = 11.3 *μ*g/ml) and BxPc-3 cells (IC_50_-24 h = 1.2 *μ*g/ml, IC_50_-48 h = 1.0 *μ*g/ml). Therefore, BxPc-3 cells are most sensitive to the PTE as well as PA, DPA and PPAC treatment (Fig. 2). PA demonstrates the strongest activity against BxPc-3 cells with IC_50_ 0.26 *μ*M (24 h), IC_50_ 0.29 *μ*M (48 h), IC_50_ 0.42 *μ*M (72 h) but only partially affects proliferation of normal human pancreatic duct epithelial cell line HPDE-6 with IC_50_ 41.6 *μ*M (24 h), IC_50_ 34.9 *μ*M (48 h) and IC_50_ 76.0 *μ*M (72 h). Moreover, PTE, PA, DPA and PPAC treatment do not affect viability of BxPc-3 cells (Fig. 2), suggesting cytostatic effect of these *P. cocos* triterpenes on BxPc-3 cells.

### Effect of PTE and PA on the cell cycle and invasive behavior of BxPc-3 cells

**Figure 3:**
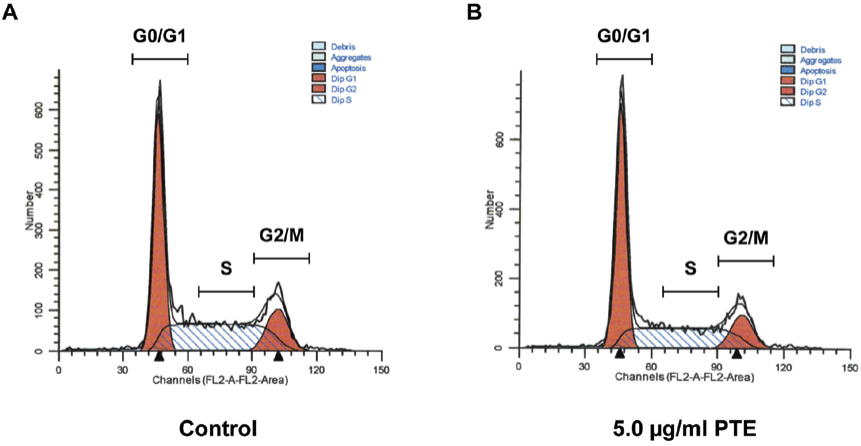
Effect of PTE on cell cycle distribution. BxPc-3 cells were treated with (A) vehicle or (B) PTE (5.0 *μ*g/ml) for 24 h. Cell cycle analysis was performed as described in Materials and methods. Representative image is shown.

**Figure 4:**
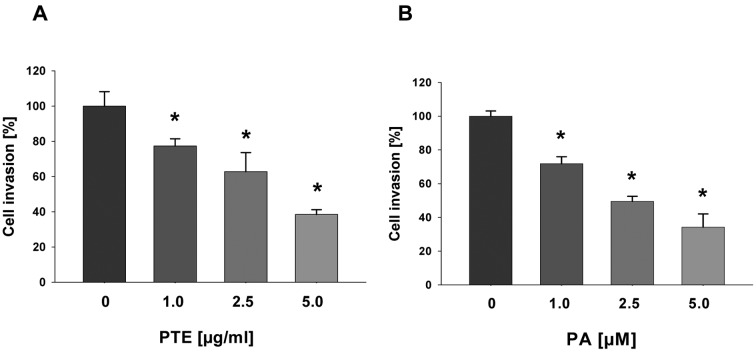
Effect of PTE and PA on invasive behavior of BxPC-3 cells. Invasion of BxPc-3 cells through Matrigel was determined after 24 h of incubation in the presence of (A) PTE (0–5.0 *μ*g/ml) or (B) PA (0–5.0 *μ*M) as described in Materials and methods. Each bar represents the mean ± SD of three experiments. Statistical analysis by ANOVA. ^*^P<0.05.

In order to evaluate whether the cytostatic effect of PTE on pancreatic cancer cells is associated with the cell cycle arrest, BxPc-3 cells were treated with PTE as described in Materials and methods. Cell cycle analysis revealed that PTE induces significant cell cycle arrest at G0/G1 phase from 44.15% (control) to 47.66% (5.0 *μ*g/ml) (Fig. 3 and Table I). To evaluate whether PTE and PA suppress invasive behavior of pancreatic cancer cells, BxPc-3 cells were treated with PTE (0–5.0 *μ*g/ml) and PA (0–5.0 *μ*M) for 24 h and cell invasion was determined as described Materials and methods. As seen in Fig. 4, both PTE and PA markedly inhibit cell invasion through Matrigel. Together, our data indicate that triterpenes from *P. cocos* not only inhibit cell proliferation through cell cycle arrest at G0/G1 phase but also inhibit invasive behavior of BxPc-3 cells.

### Triterpenes from P. cocos downregulate MMP-7 expression in BxPc-3 cell line

**Figure 5:**
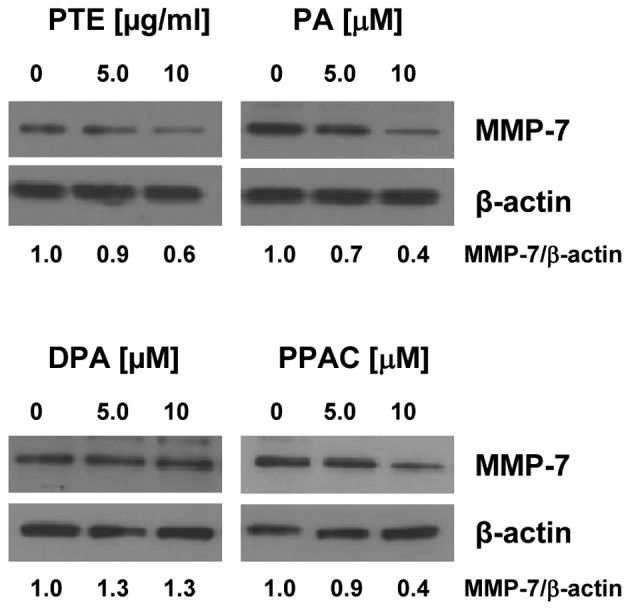
Effect of PTE, PA, DPA and PPAC on the expression of MMP-7 in BxPc-3 cells. BxPc-3 cells were treated with PTE (0–5.0 *μ*g/ml), PA (0–5.0 *μ*M), DPA (0–5.0 *μ*M) or PPAC (0–5.0 *μ*M) for 24 h and the expression of MMP-7 was evaluated by western blot analysis as described in Materials and methods. Similar results were obtained in at least two additional experiments.

To identify possible molecular targets of triterpenes from *P. cocos*, we treated BxPc-3 cells with PTE and performed DNA-microarray analysis using Array Human Pancreatic Adenocarcinoma genes as described in Materials and methods. In addition to *KRAS* (0.74±0.02, P<0.05 vs. control), PTE also markedly suppresses expression of *MMP-7* (0.67±0.14, P<0.05 vs control) (Table II). Since *MMP-7* is significantly overexpressed in pancreatic cancer samples when compared to pseudotumoral chronic pancreatitis (23), we further determined if PTE, PA, DPA and PPAC inhibit MMP-7 at the translation level as well. Western blot analysis shows that in addition to PTE and PA, PPAC inhibits protein expression of MMP-7 in BxPc-3 cells, whereas DPA has no effect (Fig. 5).

### Gene silencing of MMP-7 inhibits invasive behavior of BxPc-3 cell line

**Figure 6:**
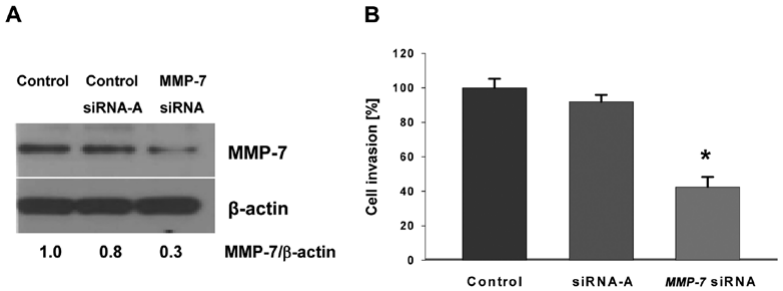
*MMP-7* gene silencing suppresses invasiveness of BxPc-3 cells. BxPc-3 cells were transfected with scrambled siRNA (siRNA-A) or *MMP-7* siRNA as described in Materials and methods. (A) Western blot analysis of MMP-7 was evaluated as described in Fig. 5. (B) Invasion of BxPc-3 cells through Matrigel was determined as described in Fig. 4. Each bar represents the mean ± SD of three experiments. Statistical analysis by ANOVA. ^*^P<0.05.

To determine whether invasive behavior of BxPc-3 cells is associated with the expression of *MMP-7*, we silenced *MMP-7* with siRNA as described in Materials and methods. As shown in Fig. 6, knockdown of *MMP-7* inhibits cell invasion by >50% in comparison with negative control cells transfected with scrambled siRNA. These results further confirm that MMP-7 greatly contributes to the invasive behavior and its deletion limits the invasive capacity of BxPc-3 cells.

## Discussion

The present study demonstrates that triterpenes extracted from *P. cocos* suppress growth and invasive behavior of human pancreatic cancer cells and only slightly affecting normal pancreatic cells. Interestingly, invasive BxPc-3 cells are the most sensitive to the treatment of a characterized triterpene mixture (PTE) as well as purified triterpenes PA, DPA and PPAC (Fig. 1). Here we show that PA is the most potent triterpene responsible for the anticancer activity of the PTE mixture (containing 55.7% PA, 31.7% DPA and 4.1% PPAC) because PA inhibits proliferation of BxPc-3 cells with the IC_50_ value (0.26 *μ*M) when compared to DPA (1.02 *μ*M) and PPAC (21.76 *μ*M).

To elucidate molecular mechanisms related to the inhibition of invasiveness of pancreatic cancer cells we used BxPc-3 cells. This cell line is the only Smad4-deficient among the four pancreatic cancer cell lines we investigated (24,25). Moreover, the loss of Smad4 is associated with a higher likelihood of metastasis, poor outcome following surgical resection (26) and predict a worse prognosis in patients with pancreatic cancer (27). Here we demonstrate, for the first time, that both PTE and PA significantly suppress invasive behavior of BxPc-3 cells. Evidently, this suppression is correlated with downregulation of MMP-7 expression. MMP-7 is overexpressed in pancreatic cancer (28), correlates with decreased survival (29,30) and contributes to cancer progression by supporting tumor size and metastasis *in vivo*(31). Therefore, MMP-7 is a suitable target involved in disease progression and the downregulation of MMP-7 expression by PA may be a useful strategy for pancreatic cancer metastasis intervention. Interestingly, PA also suppressed invasiveness through the inhibition of expression another matrix metalloproteinase, MMP-9 in breast cancer cells (12).

As recently demonstrated, PA was detected in urine and plasma of rats feed *P. cocos*(32), indicating that PA can be easily absorbed into blood. Therefore, the bioavailability of PA further promotes employment of PA or other *P. cocos* triterpenes for the treatment of different cancers including pancreatic cancer.

In conclusion, our study provides new evidence that the mixture or purified triterpenes extracted from mushroom *P. cocos* inhibits growth and invasiveness of pancreatic cancer cells. Moreover, we identified MMP-7 as a target of *P. cocos* triterpenes in pancreatic cancer cells. Further studies are in progress to investigate the exact mechanism of the inhibition of MMP-7 expression and the evaluation of the anticancer and anti-metastatic activity of PA *in vivo*.

## Figures and Tables

**Table I:** Effect of PTE on cell cycle distribution.

PTE (*μ*g/ml)	G0/G1	S	G2/M
0	44.15±1.06	40.98±1.01	14.87±0.10
2.5	44.79±0.20	40.54±1.18	14.67±0.98
5.0	47.66±1.57^a^	38.63±1.46	13.70±1.04

Cell cycle analysis was performed as described in Materials and methods. Cell cycle distribution G0/G1, S and G2/M phase in %. The data are mean ± SD from three experiments. Statistical significance;

a P<0.05.

**Table II:** Effect of PTE on the expression of human pancreatic adenocarcinoma genes.

Gene	Description	Fold change
*BIRC5*	Baculoviral IAP repeat-containing 5 (survivin)	0.85±0.12
*CCNB1*	Cyclin B1	0.87±0.03
*HSP90AA1*	Heat shock protein 90 kDa α (cytosolic), class A member 1	0.89±0.02
*IL-6*	Interleukin-6	0.86±0.04
*KRAS*	V-Ki-ras2 Kirsten rat sarcoma viral oncogene homolog	0.86±0.04^a^
*MMP-7*	Matrix metalloproteinase-7	0.67±0.14^a^
*MMP-9*	Matrix metalloproteinase-9	0.79±0.08
*RELB*	Transcription factor RelB	0.79±0.10
*TGFB3*	Transforming growth factor β-3	0.82±0.02

DNA-microarray analysis was performed as described in Materials and methods. BxPc-3 cells were treated with PTE (0 and 5.0 *μ*g/ml) for 24 h. Data are the means ± SD (n=3). Fold change PTE vs. control. Statistical significance.

a P<0.05.
